# Pros and cons of a prion-like pathogenesis in Parkinson's disease

**DOI:** 10.1186/1471-2377-11-74

**Published:** 2011-06-20

**Authors:** Ruediger Hilker, Jonathan M Brotchie, Joab Chapman

**Affiliations:** 1 Department of Neurology, Goethe University, Frankfurt/Main, Germany; 2 Toronto Western Research Institute, Toronto Western Hospital, Toronto, Canada; 3 Department of Neurology, Sheba Medical Center, Tel Aviv University, Sackler Faculty of Medicine, Israel

## Abstract

**Background:**

Parkinson's disease (PD) is a slowly progressive neurodegenerative disorder which affects widespread areas of the brainstem, basal ganglia and cerebral cortex. A number of proteins are known to accumulate in parkinsonian brains including ubiquitin and α-synuclein. Prion diseases are sporadic, genetic or infectious disorders with various clinical and histopathological features caused by prion proteins as infectious proteinaceous particles transmitting a misfolded protein configuration through brain tissue. The most important form is Creutzfeldt-Jakob disease which is associated with a self-propagating pathological precursor form of the prion protein that is physiologically widely distributed in the central nervous system.

**Discussion:**

It has recently been found that α-synuclein may behave similarly to the prion precursor and propagate between cells. The post-mortem proof of α-synuclein containing Lewy bodies in embryonic dopamine cells transplants in PD patient suggests that the misfolded protein might be transmitted from the diseased host to donor neurons reminiscent of prion behavior. The involvement of the basal ganglia and brainstem in the degenerative process are other congruencies between Parkinson's and Creutzfeldt-Jakob disease. However, a number of issues advise caution before categorizing Parkinson's disease as a prion disorder, because clinical appearance, brain imaging, cerebrospinal fluid and neuropathological findings exhibit fundamental differences between both disease entities. Most of all, infectiousness, a crucial hallmark of prion diseases, has never been observed in PD so far. Moreover, the cellular propagation of the prion protein has not been clearly defined and it is, therefore, difficult to assess the molecular similarities between the two disease entities.

**Summary:**

At the current state of knowledge, the molecular pathways of transmissible pathogenic proteins are not yet fully understood. Their exact involvement in the pathophysiology of prion disorders and neurodegenerative diseases has to be further investigated in order to elucidate a possible overlap between both disease categories that are currently regarded as distinct entities.

## Background

Parkinson's disease (PD) is a sporadic or familial neurodegenerative disorder histopathologically characterized by intraneuronal protein aggregates staining positive for α-synuclein (Lewy Bodies, LB). Concomitant Alzheimer disease type neuropathological features with amyloid deposits and plaques are also frequently found [1]. A recent clinico-pathological study on 242 brain donors with pathologically verified PD showed the presence of neurofibrillary tangles in 47% of cases with non-tremor dominant PD, neocortical amyloid plaque formation in 62% and amyloid angiopathy in 22% [2]. Although the cause of cell death in PD is unknown, the abnormal processing of neuronal proteins seems to play a key role in the pathophysiology of the disease. It has been shown that impaired proteolytic cleavage and degradation of misfolded proteins lead to their intracellular accumulation and conglomeration as LB [3]. One of the key proteins in this process is α-synuclein (SNCA). Recent *in vitro *findings demonstrated that elevated levels of misfolded SNCA can promote its self-aggregation with amyloid formation [4,5]. Misfolded SNCA is also able to spread over adjacent neurons and to induce degenerative changes in transfected cells [4]. Moreover, two post-mortem studies showed SNCA-positive LB in embryonic dopamine cells of beforehand transplanted PD patients suggesting that the microenvironment of the host brain is able to induce abnormal protein aggregation in the transplants [6,7]. Therefore, the debate came up whether SNCA behaves like a prion protein and PD might be a prion-like disease [8], which opens up an exciting new perspective on the pathomechanisms of neurodegenerative diseases in general. However, it has to be kept in mind that PD is neither infectious nor has it been transmitted to laboratory animals. The aim of this paper is to summarize important pros and cons of the prion hypothesis of PD.

## Discussion

### Molecular biology of prion diseases

Prion proteins (PrP) are infectious proteinaceous particles devoid of nucleic acids causing animal and human brain diseases by transmitting a misfolded protein configuration through brain tissue [9]. The cellular prion protein (PrP^C^) is the endogenous and physiological form with a predominant α-helix structure, which can be found on cell membranes of many tissues. In contrast, the prion protein scrapie (PrP^Sc^) isoform, which is encoded by the chromosomal prion protein gene (PRNP), is an infectious agent with a much higher proportion of β-sheet structure and a strong tendency to aggregate in form of amyloid fibers and plaques [10]. PrP^Sc ^is able to convert normal PrP^c ^into the infectious PrP^Sc ^by a conformation change from α-helix to β-sheet structure, which is considered the key event underlying prion diseases [9]. In this scenario, PrP^Sc ^acts like a template for the conversion of PrP^c ^into nascent PrP^Sc ^constituting a self-propagating vicious cycle [11]. Prion diseases can be basically caused by three pathogenic mechanisms: 1. sporadic configuration change from PrP^c ^to PrP^Sc^, 2. genetic origin by mutations in the PRNP gene and 3. transfection of PrP^Sc ^and subsequent conversion of PrP^c ^by the ingested pathogen. The accumulation and limited proteolysis of PrP^Sc ^leads to the small molecule PrP27-30 which polymerizes into amyloid potentially capable of inducing neurodegenerative changes in brain tissue [12]. Prion diseases manifest as sporadic, genetic or infectious disorders with a variety of clinical symptoms and histopathological findings. The most common form is sporadic Creutzfeld-Jakob disease (CJD) with an incidence of nearly 5 per 1 million among individual between 60 and 74 years of age [13]. CJD is histopathologically characterized by spongiform brain degeneration and astrogliosis with amyloid plaques and positive antibody staining against PrP^Sc ^in about 10 percent of cases [14,15].

### Molecular biology of alpha-synuclein (SNCA)

Several lines of evidence point to the outstanding importance of SNCA for PD pathophysiology. SNCA has been shown to be a major component of LB as the pathological hallmark of PD patients containing characteristic protein aggregates in widespread brain areas [16,17]. Mutations of the SNCA encoding gene are associated with familial forms of PD [18]. Increased intracellular SNCA levels can arise from duplication or triplication of the wild-type protein in familial cases [19,20] or from impaired lysosomal and proteasomal clearance of the protein in sporadic PD [21]. Moreover, previous data documented neuronal degeneration by the inhibition of proteasomal protein degradation and subsequent inclusion formation [22]. Other potentially toxic effects of SNCA are the induction of endoplasmatic reticulum stress, sequestration of anti-apoptotic proteins and the formation of pores in cellular membranes [23].

### Arguments for a prion-like pathogenesis in PD

The prion-like disease hypothesis of PD is particularly based upon striking parallels in the biological properties and behavior of PrP^Sc ^and SNCA. The first one is that both have

α-helical rich conformation when bound to membranes and that both undergo a change to β-sheet structure polymerizing into amyloid fibrils when present in high concentration or in mutant form [24,25]. The second important similarity is that misfolded SNCA also promotes a self-propagating conformation change of its physiological alpha-helical isomer leading to potentially toxic protein accumulation and to interference with lysosome and proteasome function [5]. The third important parallel between SNCA and PrP^Sc ^is that both reveal a kind of transmissibility. While the infectious nature of spongiform encephalopathies is undoubtedly proven, a recent pathbreaking study demonstrated that SNCA can be directly transferred from SNCA overexpressing neurons to adjacent healthy stem cells in transgenic animals with the subsequent development of inclusion bodies and signs of neuronal degeneration [4]. Direct cell-to-cell propagation of SNCA was mediated by endocytosis of recipient cells. SNCA accumulated when lysosomes in recipient cells were inhibited. The donor-derived SNCA formed juxta-nuclear inclusion bodies and recipient cells showed signs of apoptosis (nuclear fragmentation, caspase-3 activation). A further line of evidence comes from two recent post-mortem studies showing SNCA-positive LB in embryonic dopamine cells of PD patients who had been transplanted over ten years ago [6,7]. Since the appearance of LB has never been described in embryonic neurons before, it is very likely that the microenvironment of the diseased host brain led to the development of SNCA-positive aggregates in the transplants. One possible explanation for this finding is that misfolded SNCA is transmitted from the affected host to primarily healthy donor neurons inducing the fatal chain reaction of further protein misfolding.

Recently, the staging hypothesis of PD proposed by Braak and colleagues has attracted enormous attention [26]. It assumes that a currently unknown pathogen enters the central nervous system via the olfactory epithelium and enteric nerves and reaches the olfactory bulb and the dorsal motor nucleus of the vagal nerve via antero- and retrograde transport. In later disease stages, the process spreads in a sequential ascending manner particularly in neurons rich of native SNCA, such as the enthorhinal cortex, hippocampus, amygdala, substantia nigra, insula and temporo-basal cortex. The hypothesis that misfolded SNCA acts like a prion with sequential cell-to-cell transmission offers an attractive explanation of Braak's findings. These are indeed compatible with the view of PD as a prion-like disorder resulting from increased production or insufficient degradation of toxic SNCA. In this scenario, the abnormal conversion of native to β-sheet SNCA might be triggered or supported by various drivers, such as hereditary factors, aging, oxidative stress or environmental toxins.

Finally, the similarity of genetic forms of PD and prion diseases is noticeable. The inherited forms of prion diseases base on different mutations in the prion protein gene (PRNP) on chromosome 20, which are associated with various clinical phenotypes and responsible for about 10-15% of prion disorders [27]. At the protein level, the encoded mutated PrPs seem to fold into different pathogenic conformers accounting for the phenotypic variability [28]. The E200K mutation is the most prevalent one worldwide with autosomal-dominant inheritance and incomplete penetrance leading to a genetic form of CJD with rapidly progressive dementia and myoclonus along with pyramidal, cerebellar and extrapyramidal signs [29]. Just like prion disease, most PD cases are sporadic, but also several inherited monogenetic PD forms were identified over the last decade [30]. The first reported were autosomal-dominant mutations of the SNCA gene on the long arm of chromosome 4 (PARK1) [18]. Similar to mutated PrPs, amino-acid changes in the SNCA encoded protein presumably leads to an increased tendency for intracellular aggregate formation [31]. Multiplications of the wildtype SNCA gene have been shown to induce higher SNCA blood levels [32] and to cause familial parkinsonism [20]. Although the exact physiological properties of wild-type SNCA and PrP are not yet completely understood, a gain-of-function of the mutated protein with certain importance for the neurodegenerative process in PD and in prion diseases is likely in both (see [33,34] for detailed reviews). Transgenic (PG14-EGFP) mice develop an ataxic neurological disease with aggregation of a partially protease-resistant mutant PrP [35]. A similar disease process has been demonstrated in mouse prion promoter (mPrP) A53T SNCA transgenic mice, which revealed SNCA aggregation and progressive age-dependent neurodegeneration [36,37].

### Arguments against a prion-like pathogenesis in PD

CJD is a rapidly progressive disease associated with a self-propagating pathological prion protein. The life cycle of a prion includes synthesis in the endoplasmic reticulum of many cell types in- and outside the brain, transport to the external cellular membrane, secretion and trans-synaptic transport to neighbouring neurons and astrocytes, re-entry into the cell and degredation in the lysosomal apparatus. Prion proteins travel large distances systemically in the blood stream carried by lymphocytes and through the central nervous system along axons that can be more than one meter in length [38]. Concerning neuropathological similiarities between CJD and PD, it is important to consider which specific features of prion replication are actually mimicked by SNCA.

Many disease processes, such as Alzheimer's and Huntington disease, cerebellar ataxias or serpinopathies, include the accumulation of a pathological protein, an initiation "seed" of protein aggregation, stable protein aggregates, diffusion of the pathological protein and the potential for the pathological protein to cause cell damage leading in turn to enhanced protein accumulation. It is not absolutely clear how SNCA directly causes propagation of its abnormal form. As in prion pathology, it has been hypothesized that a direct interaction exists between the pathological beta pleated sheet and the normal alpha coil form leading the latter to be transformed into pathological aggregates [8]. However, even in the classical PrP associated prion diseases the exact mechanism of propagation has not been clearly defined or replicated under physiological cell free conditions. In addition, prion accumulation in cell cultures does not seem to have the same detrimental effects, for example apoptosis, which can be observed in cultures exposed to pathological SNCA. Therefore, it is currently hardly to argue that these processes are significantly similar.

From a clinical point of view, CJD and PD are very different diseases. While PD has a slow but continuously progressive course, CJD remains latent for many years followed by a rapid and exponentially devastating phase that ends within a few months. Typical clinical manifestations of CJD, such as early occurring dementia, ataxia and myoclonus, are usually not found in PD. CJD patients have typical EEG findings, for example triphasic waves or periodic lateralized epileptic discharges, and a characteristic increase of tau and 14-3-3 protein in the CSF, which are absent in PD. A recent cross-sectional study in patients with various neurodegenerative diseases has performed direct ELISA quantification of SNCA in the CSF [39]. The mean SNCA values were significantly lower in donors with a primary synucleinopathy (PD, Dementia with Lewy Bodies) than in the other study subgroups, whereas CJD patients showed markedly elevated SNCA levels. Magnetic resonance imaging often reveals hyperintense lesions in the basal ganglia and the thalamus of CJD patients, which are lacking in PD. Furthermore, spongiform encephalopathy as the pathological hallmark of CJD is not a feature of PD, whereas LB are not present in CJD. Infectiousness is a crucial hallmark of prion diseases, which can manifest after iatrogenic procedures such as neurosurgical operations as well as corneal and dural transplantations [40], whereas this kind of transmission has never been documented in PD patients so far. Finally, disease modifying substances have been found in PD animal models in vitro, while no such agents are currently available for CJD.

## Summary

The prion-like hypothesis of PD implies a redefinition of the term prion away from its exclusive use in transmissible spongiform encephalopathies towards a general infectious principle in protein misfolding and aggregation diseases. It focuses the researchers' attention on the key role of SNCA malfunction in the pathogenesis of PD and implies that blocking the mishandling of SNCA and other proteins might be an effective therapeutic strategy in PD patients. However, at the current state of knowledge, a number of issues advise caution before categorizing PD as a prion disorder, because the molecular pathways of transmissible pathogenic proteins are not yet fully understood and clinical, brain imaging, CSF and neuropathological findings clearly differ in patients with classic prion diseases and PD.

## Competing interests

The authors declare that they have no competing interests.

## Authors' contributions

All authors participated on the debate during the 3^rd ^World Congress on Controversies in Neurology (CONy) held on October 8-11 2009 in Prague and prepared, read and approved the final manuscript. RH primarily drafted had the background section and the pro arguments in the discussion, JC primarily drafted the cons, and JMB had particular responsibility for the conclusions in the summary.

## Pre-publication history

The pre-publication history for this paper can be accessed here:

http://www.biomedcentral.com/1471-2377/11/74/prepub
